# Convergent and divergent roles of the glucose-responsive kinase SNF4 in *Candida tropicalis*

**DOI:** 10.1080/21505594.2023.2175914

**Published:** 2023-02-13

**Authors:** Cai-Ling Ke, Shi Qian Lew, Yi Hsieh, Szu-Cheng Chang, Ching-Hsuan Lin

**Affiliations:** Department of Biochemical Science and Technology, College of Life Science, National Taiwan University, Taipei, Taiwan

**Keywords:** *Candida tropicalis*, AMPK, SNF4, glucose derepression, cell wall integrity, virulence

## Abstract

The sucrose non-fermenting 1 (*SNF1*) complex is a heterotrimeric protein kinase complex that is an ortholog of the mammalian AMPK complex and is evolutionally conserved in most eukaryotes. This complex contains a catalytic subunit (Snf1), a regulatory subunit (Snf4) and a scaffolding subunit (Sip1/Sip2/Gal73) in budding yeast. Although the function of AMPK has been well studied in *Saccharomyces cerevisiae* and *Candida albicans*, the role of AMPK in *Candida tropicalis* has never been investigated. In this study, we focused on *SNF4* in *C. tropicalis* as this fungus cannot produce a *snf1Δ* mutant. We demonstrated that *C. tropicalis SNF4* shares similar roles in glucose derepression and is necessary for cell wall integrity and virulence. The expression of both *SNF1* and *SNF4* was significantly induced when glucose was limited. Furthermore, *snf4Δ* strains exhibited high sensitivity to many surface-perturbing agents because the strains contained lower levels of glucan, chitin and mannan. Interestingly, in contrast to *C. albicans sak1Δ* and *snf4Δ*, *C. tropicalis snf4Δ* exhibited phenotypes for cell aggregation and pseudohypha production. These data indicate that *SNF4* performs convergent and divergent roles in *C. tropicalis* and possibly other unknown roles in the *C. tropicalis SNF1-SNF4* AMPK pathway.

## Introduction

*Candida* species are among the most prevalent pathogenic fungi that cause serious diseases around the world, and *C. albicans* is the most common species among the 150 species [1]. Residing on the body and skin of most of the world’s population, *C. albicans* can cause severe superficial and systemic infections, such as vulvovaginal candidiasis, intestinal inflammation and thrush, mainly occurring in immunocompromised individuals [2–5]. In addition to *C. albicans*, recent studies have revealed non-*albicans Candida* species (NACs) as emerging threats leading to increasing candidiasis or candidemia cases around the world [6]. *C. tropicalis* is one of the common *Candida* species that is evolutionally close to *C. albicans*, but it is prevalent in mainly tropical areas [7, 8]. This species is known for its rapidly rising infection rates among immunocompromised individuals and exhibits high resistance to fluconazole amphotericin B [9–11], making it an unneglectable threat.

To address different conditions, *C. albicans* can utilize multiple carbon sources, which eventually trigger different signalling pathways, such as the mitogen-activated protein kinase (MAPK) and AMP-activated protein kinase (AMPK) pathways, to overcome stresses caused by abrupt environmental changes [12]. AMPK is a highly conserved protein kinase in eukaryotes and is composed of one α catalytic subunit (Snf1), β regulatory subunits (Gal83, Sip1 and Sip2) and the γ subunit (Snf4) found in *S. cerevisiae* [13–15]. This heterotrimeric protein kinase plays an indispensable role in cell energy homoeostasis, such as in metabolizing sugars and lipids, and in regulating environmental pressure [14,15]. In particular, the complex is regulated by alterations in the AMP:ATP ratio in the cell [15,16]. At high glucose levels (low AMP/ATP ratio), nonphosphorylated Snf1 is unable to activate the Mig1 protein, the most important transcriptional repressor [17,18]. The Mig1 then stays in the nucleus and binds to many carbon source responsive elements (CSREs) of *GAL3*, *HXT2*, *MAL31*, *MAL32* and *SUC2* [17]. Nevertheless, under low concentrations of glucose (high AMP/ATP ratio), Snf1 and AMPK are activated and phosphorylated. Activated Snf1 phosphorylates the transcriptional repressor Mig1, leading to translocation of Mig1p from the nucleus to the cytoplasm and allowing the cell to survive in extreme environments by utilizing nonglucose carbon sources as alternative energy sources [19].

In fungi, such as *S. cerevisiae* and *C. albicans*, the roles of Snf1 and Snf4 in AMPK activity are highly conserved [15, 20, 21, 22]. However, unlike that of budding yeast, Snf1 of *C. albicans* is an essential gene and is required for viability, as several groups failed to generate homozygous *snf1Δ* mutants [23]. Interestingly, Aaron and colleagues successfully recovered homozygous *snf1Δ* in the strain background of *mig1Δ* but not in *mig2Δ* [21]. They suggest that several essential genes are regulated by the Snf1-Mig1 glucose repression pathway or the sum of cumulative effects of low expression of Mig1-regulated genes, leading to viability [21]. Unlike *SNF1* in *C. albicans*, homozygous *snf4Δ* was able to recover [22], and phenotypes of *snf4Δ* exhibited growth defects on several nonfermentative sugars and reduced hypha formation, similar to *SAK1* deletion strains [22].

Despite the close relationship between *C. albicans* and *C. tropicalis* in many aspects, several studies have revealed that these pathogens exhibit distinct filamentous responses under different culture conditions [24–27]. Furthermore, loss of some homologous genes in the two fungi also led to distinct filamentous behaviours [27–31], suggesting that the regulatory circuit associated with hyphal development in the two *Candida* species is divergent. In this study, we focused on understanding the roles of Snf4 in *C. tropicalis*. We found that Snf4 governs several physiological aspects, including chronological life span (CLS) in low-glucose conditions, cell wall integrity and virulence. Interestingly, in contrast to *C. albicans snf4Δ*, the *C. tropicalis snf4Δ* strains promoted cell aggregation and hypha formation, suggesting a divergent function of *SNF4* in cell separation and hypha formation in the two *Candida* species.

## Materials and methods

### Media and reagents

Roswell Park Memorial Institute 1640 (RPMI 1640), YPD (yeast extract-peptone-dextrose), YPD supplemented with 200 μg/mL nourseothricin (Werner BioAgents, Jena, Germany) or 50% serum (Gibco, 1 861 237; Life Technology, Carlsbad, CA, USA) and Spider and Spider D (mannitol replaced with 0.5% glucose) were prepared for cell culture, strain construction, hyphal induction and colony morphological inspection [22, 32]. Minimal medium plates supplemented with 50 mM glucose, galactose, sucrose or glycerol were prepared for growth testing. Unless otherwise stated, media and chemicals were purchased from Sigma‒Aldrich, Merck KGaA.

### Plasmid and strain construction

The *C. tropicalis* strains and DNA oligos used here are listed in Tables 1 and 2, respectively. The plasmid pEM010 [34] was used to construct the required *C. tropicalis SNF4* mutant strain. The *C. tropicalis* strain sequence was acquired from *Candida* Genome Database (http://www.candidagenome.org/) [33]. The primers used in this experiment were synthesized by MDbio Biotech Co., Inc. (Taipei, Taiwan). The primers 1694/1695 and 1696/1697 were used to amplify the 5’ flanking and 3’ flanking DNA fragments of the *SNF4* gene by performing polymerase chain reaction (PCR). The restriction enzymes KpnI-HF/ApaI and NotI-HF/SacII were used to digest these PCR products, which were then inserted into the pEM010 plasmid to generate the pEM010-*SNF4* KO plasmid. The plasmid pEM010-*SNF4* KO was cut with KpnI/SacII and transformed into the *C. tropicalis* MYA3404 (YL477) to create heterozygous *snf4∆/SNF4*. The *SAT1* marker was removed after treatment with 1% casamino acids [34]. The heterozygous strains were retransformed with the linearized *SNF4* KO plasmid to generate the homozygous *snf4*∆ strains (YL 2039, YL2040). The primers 6/1700, 7/1701, and 1698/1699 were used to verify the *snf4*∆ genotype. To construct the *SNF4* complemented strains, the *SNF4* promoter and its open reading frame were amplified with the 1793/1794 primer pair. The PCR product was digested by KpnI/ApaI and then cloned into the pEM010 plasmid to generate the pEM010-CtSNF4 AB plasmid. The pEM010-CtSNF4 AB plasmid was digested with KpnI and SacII. The linearized products were then transformed into homozygous *snf4*∆ strains to generate the complemented strains (YL 2044, YL 2045). The primers 1698/1699 were designed and used to confirm the complemented strains.

**Table 1.** Strains used in this study.

Strain	*MTL*	Genotype	Ref.
YL477	*a/*α	Wild type (MYA3404)	[33]
YL2039	*a/*α	*snf4/snf4:SAT*	This study
YL2040	*a/*α	*snf4/snf4:SAT*	This study
YL2044	*a/*α	*snf4/SNF4:SAT1*	This study
YL2045	*a/*α	*snf4/SNF4:SAT1*	This study

**Table 2.** Primers used in this study.

No.	Sequence (5’ to 3’)
6	CTCAACCATAGCAATCATGG
7	GCGAAAAAGTGGGCACTAAG
819	TGGATTCTGGTGATGGTGTTAC
820	TCAAGTAGTCGGTCAAGTCTCT
1694	GGAGCGGGTACCTGCATCACCTTTAAAATTCCCCT
1695	GGAGCGGGGCCCTCCGTTTGGGTTTGGTGAGA
1696	GGAGCGGCGGCCGCACCCAAAATACCCAACACCAC
1697	GGAGCGCCGCGGATCCCTCGTCATTA
1698	ATTTFCTGGGTTATTAACTTCAAGTGA
1699	ATACATACCACTTTAACCAACGAC
1700	TGTCATCAAATCTAGCATGCTCA
1701	CTTATGTCGTATGTGCTATTATTTGTGG
1793	GGAGCGGGTACCGGAGAGGTCATTGCCAATATCAG
1794	GGAGCGGGGCCCGTGGTGTTGGGTATTTTGGGT
1820	TCCAGTCGTAGATGACCAAGG
1821	TGAACCCCTTCAAATTCTTCC
1854	TGGTTCCTTGTTTTCCTTGC
1855	TCACCGACACATCTCATGGT
1856	TCGTTGTCGGTGCTTATTCA
1857	GGTTGGACCTATCGTGCCTA
1899	CAGGAACGATGCGACTTGTA
1900	TCGACGGTATTCATCCCAAT
1901	TTGCTGGAAAGGCAGAAAT
1902	GGTTTTGGCTCATCACCTTC
1903	CGAAACCATTGAAATCCCTTT
1904	CGGCCTTTTTCTTATCGTCA
1905	TTGGTTCTTTGGCTTTCTGG
1906	GAATGGACTGGAGCATCACC
1907	CCAGAAGAATTGAAAACTGAACC
1908	TAACGGCCTTGGATGTTGAT
1909	CGTTGCTTGGGTTGGTTTAT
1910	GCACCACCATCACCTTCTTT
1911	CTTCCGACCCAGACAACTTC
1912	TGGAACACAAGTGGGCAATA
1913	CAGGGGTTGATCCTCGTAAA
1914	TTGAACGCTGACATTTGGAG
1915	TGCTACTGGTTCTGCTCCAA
1916	GGTGGTGGTGGTGGTAAAAC
1917	AATCATCCCAATCGCAATCT
1918	CCTGGTTGGCTGTATTCTCC
1923	CAAATCGACCAACGAGGTTT
1924	GGCGACAGTGACAAGAATGA
2510	TGAAGACTAACTACTGCGAAAG
2511	GTTAAGACTACGACGGTATCTG
2520	CACCCATGATGTTCAAGCAC
2521	CGGCTTGATCAACAATTTCA
2522	GGTCCAGGTGATGCTTTCAT
2523	CGCAATGGGTAGACATGTTG
2524	ATCTTTGGCATTGCATTGGT
2525	TGTCCCAATTGAAGAAGGAAA
2528	CTTTGGGATGAAGGATTCCA
2529	TTTGTTCACGAGCAATCCAG
2530	GACCCACTGCCTGAATTGTT
2531	TGATGCCTCGTAGAATGCTG
2532	ACAATGGTGCCTCATCAACA
2533	TCCACCACAAAGTTTCACCA
2534	AGACCTGATTGGGCTGATTG
2535	CCAGGACCTGTCCAATTCAT
2536	TGGTGAGCTTACTGCACCAG
2537	TGACCAGGGTGATTAGCACA
2538	AAAGCATCCAATGCTGGTTC
2539	CTGCCGGATTTACATCCACT
2546	ATTGTTGCCATGACTGGTGA
2547	CAGCTTCTTTGGCAACATCA

### Growth tests

Overnight cultures of *C. tropicalis* cells were adjusted with sterilized ddH_2_O to an OD_600_ = 1.0 and cultivated in a conical flask at 30°C and 125 rpm. The concentration of the cell culture was measured at 0, 2, 4, 6, 8, 10, 12, 16, 24, 36, 48 and 72 hours.

### Cell sedimentation test

Overnight cultures of the *C. tropicalis* wild-type strain, *SNF4* deletion mutants and complemented strains in YPD were placed on a glass holder rack for 10 min to evaluate cell sedimentation. Cells of each precipitated sample with and without vortexing were observed using the Eclipse Ti inverted microscope (Nikon Instruments Inc., Melville, NY, USA) to determine cell morphology.

### Chronological lifespan spotting assay

Wild-type and *snf4*∆ *C. tropicalis* cells were cultured in minimal medium supplemented with 2% glucose and 0.5% glucose (low glucose) for 40 days as described previously [35, 36]. Every 2 or 4 days, 200 μL of each culture was serially diluted 10-fold to a 10^−5^ dilution, and 2 μL of each dilution was subsequently spotted onto minimal medium plates containing 2% and 0.5% glucose, respectively. The plates were incubated at 30°C. Growth was assessed every 2 or 4 days, and images were obtained.

### Sensitivity assays

Overnight cultures of wild-type and *snf4*∆ *C. tropicalis* and complemented strains were resuspended in sterilized ddH_2_O to OD_600_ = 1.0 and serially diluted 10^−5^. Two microlitres of each sample was spotted onto minimal medium plates containing different carbon sources, YPD or RPMI 1640 with or without 0.02% SDS, 0.8 μM calcofluor white, 0.8 μg/mL caspofungin or 256 μg/mL fluconazole and then incubated at 30°C for 2–3 days prior to imaging.

### Colony phenotypic assays

Colony morphologies on different agar media were tested by dilution spot assays as described previously [22]. Overnight cultures of the wild-type, *snf4*∆ and complemented strains were appropriately diluted and spotted on YPD agar with or without 50% foetal calf serum, Spider medium and Spider D medium and incubated at 30°C for 5 days before imaging.

### Hypha formation assays

Overnight cultures of the wild-type MYA3404, *snf4*∆ and complemented strains of *C. tropicalis* were resuspended fresh YPD medium containing 0% serum or 50% serum or RPMI 1640 liquid medium for 6 hours. Hyphal formation was evaluated using the Eclipse Ti inverted microscope (Nikon Instruments Inc., Melville, NY, USA). Four fields were assessed, and at least 300 cells were counted for every *C. tropicalis* strain. The hyphal formation ratio was measured with NIS-Elements BR software.

### Calcofluor white staining

The staining was performed according to the established protocol [37]. Briefly, 1 drop of 1% calcofluor white and 1 drop of 10% potassium hydroxide (KOH) were added and incubated for one minute at 25°C. Samples were examined with an Eclipse Ti inverted microscope.

### Measuring the cell wall carbohydrate content

The total carbohydrate content of the cell wall was extracted using the phenol/sulphuric acid method, as previously described [38, 39]. Briefly, samples were disrupted with glass beads and centrifuged. Glucan, mannan and chitin were quantified by the established protocol as previously described [38, 39]. Briefly, pellets were subjected to H_2_SO_4_ hydrolysis, boiled at 100°C and neutralized with Ba(OH)_2_ [38, 39]. HPAEC-PAD (high-performance anion-exchange chromatography with pulsed amperometric detection) was conducted with a Dionex Bio-LC5000 system (Thermo Fisher Scientific). Glucose, mannose and glucosamine monosaccharides were used as standards and represented the glucan, mannan and chitin cell wall polysaccharides, respectively. The addition of exogenous galactose during extraction served as an internal control [38, 39].

### Quantitative reverse transcription PCR

*C. tropicalis* cells of the wild-type, *snf4*∆ and complemented strains were revived with 10 mL of YPD liquid medium at 30°C for an additional four hours. Cells were harvested by centrifugation and total RNA was harvested to detect *NRG1* and *Ume6* expression. To detect *SNF1* and *SNF4* expression, cells were harvested, washed with PBS three times and placed in ddH_2_O containing 2%, 0.5% or 0.1% glucose, glycerol, sucrose or galactose for 20 min. Then, total RNA was harvested from the cells. RNA extraction was performed according to the MasterPureTM Yeast RNA Purification Kit protocol (Epicentre, Madison, WI, USA) and involved treatment with DNase I (Thermo Fisher Scientific, Waltham, MA, USA) to remove DNA. An iScriptTM cDNA Synthesis Kit (Bio-Rad Laboratories., Inc., Hercules, CA, USA) was used to synthesize complementary DNA (cDNA). Quantitative PCR was conducted in a BioRad CFX Manager system (Bio-Rad Laboratories, USA). The primer pairs for the detection of *ACT1*, *RND18*, *SNF1*, *SNF4*, *MNS1*, *CWH41*, *ROT2*, *MNN1*, *MNN14*, *MNN2*, *MNN5*, *OCH1*, *PMT1*, *PMT2*, *PMT4*, *PMT5*, *MNT1*, *MNT2*, *PMR1*, *FKS1*, *FKS2*, *CHS1*, *CHS3*, *NRG1*, and *UME6* were 819/820, 2510/2511, 1923/1924, 1820/1821, 2532/2533, 2528/2529, 2530/2531, 2536/2537, 1899/1900, 1901/1902, 2538/2539, 2534/2535, 1909/1910, 2520/2521, 2522/2523, 2524/2525, 1905/1906, 1907/1908, 2546/2547, 1854/1855, 1856/1857, 1911/1912, 1913/1914, 1915/1916 and 1917/1918, respectively (Table 2). Gene expression was normalized to that of *ACT1* and *RND18*, respectively. The data were analysed by Student’s t-test. All experiments were repeated three times independently.

### Biofilm assays

The biofilm biomass in a silicone model of *C. tropicalis* was determined by the established protocol as previously described [40, 41]. Preweighed sterilizing silicone squares (Bentec Medical, PR72034-06N) were first incubated in bovine serum (Gibco, 1861237; Life Technology) overnight at 37°C with gentle shaking in 12-well polystyrene plates. The silicone squares were then washed PBS (2 mL) three times and transferred into 2 mL of RPMI 1640 medium or synthetic urine for adhesion. Approximately 2 × 10^7^ *C. tropicalis* cells from overnight culture in YPD medium were carefully placed on top of each silicone and incubated at 37°C for 4 hours with gentle shaking to allow cell adhesion. Each silicone square was then washed with PBS three times and placed in fresh Spider medium for 24 hours at 37°C with shaking at 100 rpm. The supernatants were removed, and the silicone squares were dried overnight. The amount of biofilm mass formed on the silicone was determined by weighing. The results were analysed by Student’s t-test, and all experiments were repeated three times independently.

### Virulence assay using the greater wax moth (*G. mellonella*) model and fungal burden assessment

Ten larvae were used in each group to determine the pathogenicity of each strain [42]. Overnight cultures of *C. tropicalis* cells in YPD medium were harvested and washed three times with PBS. *C. tropicalis* cells of the wild-type MYA3404, *snf4*∆ and complemented strains (2.25 × 10^6^ cells/10 µL) were injected into a greater wax moth. The virulence assay was monitored for 7 days to determine the survival rates. To quantify *C. tropicalis* colony-forming units (CFUs) in *G. mellonella* haemolymph, a protocol established with *C. albicans* was followed, as described previously (Rossoni et al., [43]. Briefly, each 24-hr infected larva was cut using a sterilized blade and squeezed to collect the haemolymph (~100 µL/per larva) in a sterilized tube. Samples were then homogenized, serially diluted and plated on YPD containing chloramphenicol (100 μg/mL). The plates were incubated at 30°C for 2 days to determine the number of colony-forming units (CFU/mL) for each larva.

### Virulence assays using ICR mice and fungal burden assessment

Male ICR (Institute for Cancer Research) mice (four to five weeks old) purchased from BioLASCO (Taiwan) were tested (*n* = 9 for each group) [25, 44]. Overnight cultures of *C. tropicalis* cells in YPD medium were harvested and washed with PBS three times. *C. tropicalis* cells of each strain (5 × 10^6^ cells/200 μL) were injected through the tail vein. The virulence assay was monitored for 30 days to determine the survival rates. A *P* value less than 0.05 was considered statistically significant. To assess fungal burden, *C. tropicalis*-infected mice were sacrificed on the third day post-infection. The brains, spleens and kidneys (*n* = 5/each group) of the mice were removed, weighed, and placed in 10 mL of PBS to be homogenized (30,000 rpm for 2 min) with IKA dispersers (IKA, T10 basic ULTRA-TURRAX® Crushing Disperser, Germany). The homogenized samples were serially diluted and placed on YPD containing chloramphenicol (100 μg/mL). Plates were incubated at 30°C for 2 days to determine the number of CFUs per gram of each organ tissue homogenate. All experiments were performed in accordance with animal protocols approved by the IACUC (Institutional Animal Care and Use Committee) at NTU (National Taiwan University) (approval #00228).

### Statistical analyses

Results were considered statistically significant when the *P* value was less than or equal to 0.05. Statistical analyses of virulence assays were conducted with the log-rank test, while others were evaluated by Student’s t-test.

## Results

### The *SNF4* gene is involved in the utilization of nonglucose carbon sources for metabolism in *Candida tropicalis*

**Figure 1.**
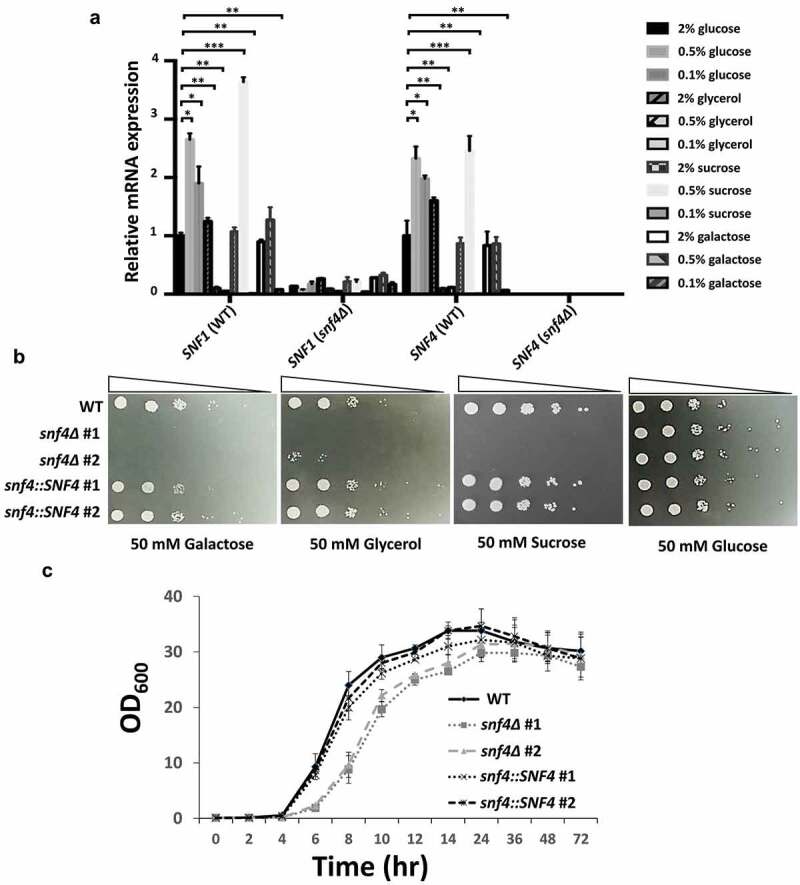
*C. tropicalis* Snf1-Snf4 AMPK is involved in glucose derepression. (a) Low glucose levels (0.5% and 0.1%) and sucrose (0.5%), but not high glucose (2%) and other nutritional conditions, induced *SNF1* and *SNF4* expression. *SNF4* deletion showed a significant reduction in *SNF1* expression under different sugar treatments, indicating that *SNF4* is required for AMPK activation and that the activated pathway is necessary for utilizing nonglucose carbon sources. (b) Deletion of *C. tropicalis SNF4* completely abolished the ability to utilize nonglucose carbon sources. (c) the monitored growth rates showed mild growth defects in *snf4δ* strains compared to the wild-type and complemented strains. The values are the means ± SDs from three experimental replicates. *, *P*<0.05; **, *P*<0.01; ***, *P*<0.001.

Previous studies have shown that the activated AMPK pathway plays a primary role in glucose derepression in both *S. cerevisiae* and *C. albicans* [15, 20, 21, 22]. We first evaluated the expression of AMPK component genes under different concentrations of glucose and other carbon sources in *C. tropicalis*. As shown in Figure 1(a), the expression of both *SNF1* and *SNF4* increased significantly under low glucose conditions (0.5% and 0.1% glucose) compared to normal culture conditions (2% glucose). Furthermore, the *SNF1* and *SNF4* genes were not induced after glycerol (2%), sucrose (2%) and galactose (2% and 0.5%) treatments. Nevertheless, we observed that the expression of both *SNF1* and *SNF4* was highly induced after 0.5% sucrose treatment (Figure 1(a)). It was reported that replacement of glucose with sucrose resulted in an elevated AMP:ATP ratio, leading to activation of the Snf1-Snf4 AMPK pathway and phosphorylation of Mig1 [19,45]. Interestingly, unlike the 0.1% glucose treatment group, replacement of glucose with 0.5% or 0.1% glycerol, 0.1% sucrose or 0.1% galactose almost shut down *SNF1* and *SNF4* expression (Figure 1(a)), suggesting that the production of metabolic signals (AMP:ATP ratio) with different concentrations of carbon sources that trigger glucose derepression in *C. tropicalis* remains unclear and requires further investigation. Undeniably, these data indicate that *C. tropicalis* the *SNF1-SNF4* AMPK pathway is an AMP:ATP ratio-dependent pathway and plays a similar role in glucose derepression in budding yeast and *C. albicans* [15,20–22].

Unlike in budding yeast, *SNF1* is essential for *C. albicans* survival [23] unless the major transcriptional repressor Mig1 is absent [21]. Correspondingly, several unsuccessful attempts have been made to recover *snf1∆* in *C. tropicalis*, suggesting that the role of *C. tropicalis SNF1* in cell viability might be the same as that of *C. albicans SNF1*. Thus, *C. tropicalis SNF4* mutant strains were constructed to understand whether *C. tropicalis* AMPK plays different roles than that in *C. albicans*. First, *snf4∆* exhibited significant reductions in *SNF1* expression in all of the abovementioned culture conditions (Figure 1(a)), indicating that Snf4 is required for AMPK and Snf1 function in *C. tropicalis*. Furthermore, tests of the sensitivity to different carbon sources, including glucose, sucrose, galactose and glycerol, showed that the *C. tropicalis* wild-type strain exhibited normal growth on all of the plates mentioned above, while the *snf4*∆ strains spotted on sucrose, galactose and glycerol plates exhibited severe growth defects (Figure 1(b)). The *SNF4* complemented strains showed recovery of the growth defects in the plates mentioned above (Figure 1(b)). Furthermore, the cell growth showed that the *snf4* mutant strains exhibited some growth defects in YPD liquid medium (Figure 1(c)). These results indicate that a convergent role of AMPK in both *C. albicans* and *C. tropicalis* is required for maintaining normal physiological function and for utilization of nonglucose carbon sources when glucose is not sufficient. We hypothesize that *C. tropicalis snf4∆* might also fail to activate the Snf1p-Mig1/Mig2 AMPK pathway, in which the nonphosphorylated Mig1/Mig2 is retained in the nucleus and binds to many CSREs of nonfermenting sugar genes, leading to repression of their expression, making it unable to utilize nonglucose carbon sources as alternative energy sources [20, 21, 22]. These results indicate that survivability under glucose starvation of *C. tropicalis* is highly dependent on Snf1-Snf4 AMPK.

### The *SNF4* gene is involved in the regulation of chronic life span (CLS) in *Candida tropicalis*

**Figure 2.**
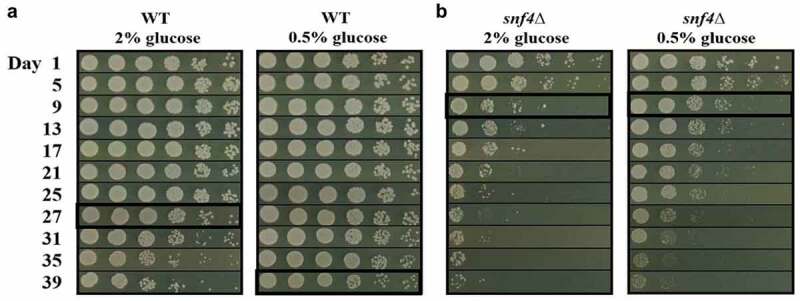
*C. tropicalis SNF4* is necessary for chronological life span (CLS) and life extension when glucose is insufficient. (a) CLS assay of the wild-type strain grown under restricted (0.5% glucose) conditions exhibited longer CLSs than those grown under nonrestricted (2% glucose) conditions (black box). (b) the *SNF4* deletion strain had the same CLS under both 2% and 0.5% glucose growth conditions and exhibited a significant reduction in CLS (black box) compared to the wild type (panel A).

As the population continues to age, reducing the occurrence of ageing-related diseases has become an important issue. Studies have proven that caloric restriction (CR) can extend the ageing process in various eukaryotic cells and animals through the AMPK signalling pathway [36,46–48]. In budding yeast, CR can activate the Snf1 protein kinase by regulating the yeast’s two-phase growth (Diauxic shift), thereby extending its lifespan [35, 36]. Whether AMPK is involved in the regulation of CLS in *Candida* spp. has never been investigated. Therefore, we aimed to understand whether AMPK is involved in the regulation of the CLS of *C. tropicalis*. Since glucose is the main energy source for most microorganisms, in this experiment, 2% glucose medium was used as the control, and 0.5% glucose medium was used to simulate CR and activate AMPK based on our previous data (Figure 1(a)). Figure 2(a) shows that when wild-type *C. tropicalis* was grown on 2% glucose, it began to show growth defects on the 27^th^ day, while under CR conditions, a mild growth defect was shown until the 39^th^ day. These results indicate that the CLS of *C. tropicalis* can be extended under restricted calorie intake. In addition, *snf*4∆ strains on both media exhibited slow growth (Figure 2(b)) compared to the wild-type strain (Figure 2(a)). The data suggested that the inability of the AMPK-defective strains to sense glucose could consistently force the transcriptional repressor Mig1 to be retained in the nucleus, leading to the inhibition of many genes required for maintaining physiological function in *C. tropicalis*. In addition, regardless of whether the *snf*4∆ strains were cultured with 2% or 0.5% glucose medium, they began to show growth defects after 9 days of incubation (Figure 2(b)). These results imply that AMPK regulates CLS and is required for life extension during CR in *C. tropicalis*.

### *SNF4* gene is involved in the regulation of *Candida tropicalis* cell wall integrity

**Figure 3.**
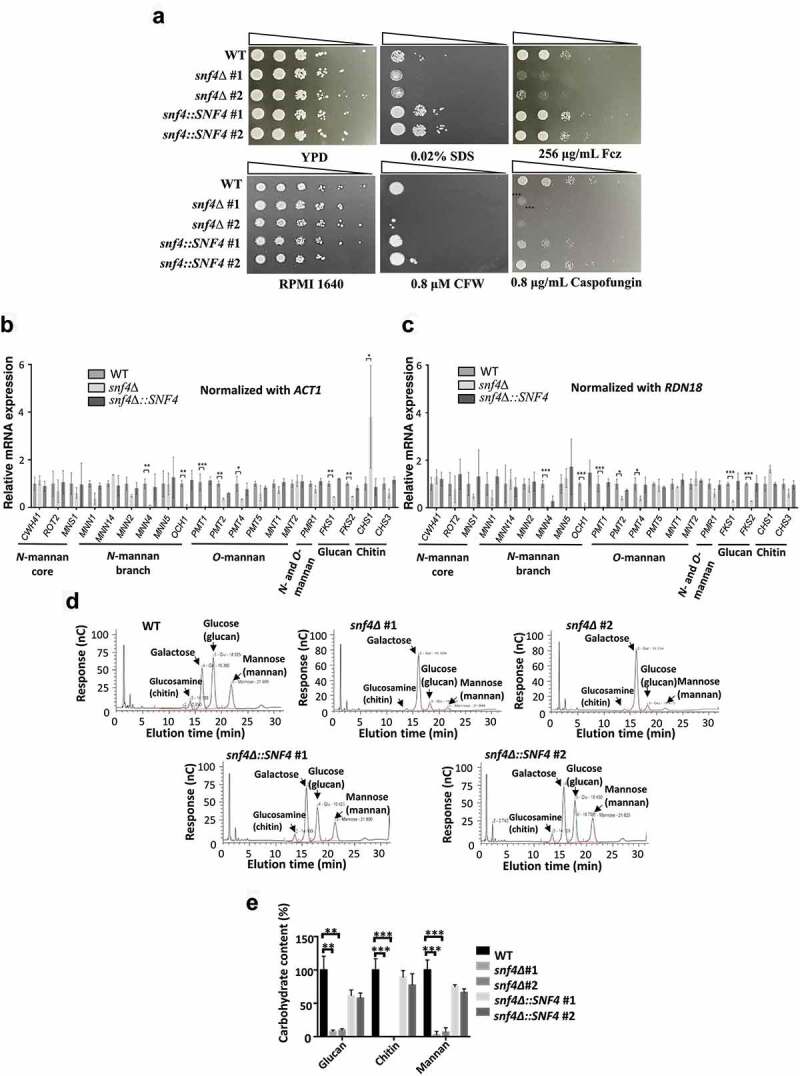
*SNF4* deletion impaired cell wall integrity, leading to sensitivity to cell surface-perturbing agents. (a) the *snf4δ* strains showed increased sensitivity to cell surface-perturbing agents and antifungal drugs. Fcz: fluconazole; CFW: calcofluor white. Analyses of *SNF4*-regulated genes in the synthesis of fungal cell wall polysaccharides after normalized with (b) *ACT1* and (c) *RND18*. The data showed that *snf4δ* caused a significant reduction in the expressions of some *N*-linked amd *O*-lined genes, and *FKS1* and *FKS2*. (d) Representative images of the cell wall proportion of each sample determined by HPAEC-PAD. Galactose was added as an internal standard to control the extraction process. (e) Quantitative determination of the proportion of beta-glucan, chitin and mannan via HPAEC-PAD showed considerable decreases in glucan, chitin and mannan levels in the *snf4δ* strains. The values are the means ± SDs from three experimental replicates. *, *P*<0.05; **, *P*<0.01; ***, *P*<0.001.

Glucose is the building block for other sugar derivatives and can undergo a series of metabolic conversions and participate in the synthesis of cell walls [49]. In addition, AMPK is closely associated with glucose sensing and nonglucose carbon source utilization, and AMPK-deficient strains of both *S. cerevisiae* and *C. albicans* are sensitive to many cell surface-perturbing agents [15, 20, 21, 22]. Thus, cell membrane-perturbing (fluconazole and SDS) and cell wall-perturbing (calcofluor white and caspofungin) agents were selected for further use in sensitivity assays [50, 51]. Figure 3(a) shows that the growth of *snf4*Δ revealed marked impacts on drug and stress susceptibility compared to the wild-type strain. Resistance to these stress agents was restored in the *SNF4-*complemented strains. These data are consistent with those for the *C. albicans snf4*∆ strains [22]. To further examine whether the cell wall integrity of *C. tropicalis* is affected by the deletion of *the SNF4* gene, the cell wall polysaccharide concentration of *C. tropicalis* via was analysed by the phenol‒sulphuric acid method [38]. To further understand how *SNF4* regulates cell wall integrity, an analysis of gene expression was performed on the genes involved in the synthesis of fungal cell wall polysaccharides such as mannan, β-glucan, and chitin. The mannoprotein is synthesized by mannosyltransferase, which is encoded by *MNN* family genes; β-glucan is synthesized by β-glucan synthase, which is encoded by *FKS* family genes; and chitin is synthesized by chitin synthase, which is encoded by *CHS* family genes [52–54].

Several genes involved in mannosylation, cell wall, hyphal growth or virulence in *C. albicans* have been summarized in the review article [55], Three N-mannan core structure genes (*MNS1*, *CWH41* and *ROT2*), six N-mannan branch genes (, *MNN1*, *MNN14*, *MNN2*, *MNN4*, *MNN5* and *OCH1*), six O-linked related genes (*PMT1*, *PMT2*, *PMT4*, *PMT5*, *MNT1*, *MNT2*,), one gene (*PRM1*) that are involved in both the N- and O-linked mannan structure were therefore selected. Furthermore, two glucan synthesis genes (*FKS1, FKS2*), and two chitin synthase genes (*CHS1* and *CHS3*) were selected for the analyses. Based on the reference [56], two related stable genes (*ACT1* and *RND1*) were chosen as internal controls to evaluate gene expressions for the cell wall. As shown in Figure 3(b,c), the expression levels of most *N*-mannan core structure and branch genes in the mutant strain were similar to those in the wild-type strain except that *MNN4* (*MNN6* regulator) [57] and *OCH1* (initiation of α 1,6-mannose) [58] were significantly reduced in the mutant strain after the gene was normalized with *ACT1* and *RDN18* [56]. Three *O*-linked related genes (*PMT1*, *PMT2* and *PMT4*), which are involved in the initiation of *O*-glycosylation, reduced in the mutant strain [59–61]. Furthermore, *CHS1* showed a significant increase when normalized with *ACT1* (Figure 3(a)), although no statistically significant difference was observed between the wild-type and *snf4Δ* when normalized with *RND18* (Figure 3(b)). Nevertheless, the expression levels of both *FKS1* and *FKS2* decreased significantly in the *snf4Δ*. This result indicated that *SNF4* gene deletion could affect some *N*- and *O*-linked glycosylation structures and severely reduce the synthesis of β-glucan in the *C. tropicalis* cell wall by regulating the gene expression of *FKS1* and *FKS2*. The lower expression of *FKS* genes in the *snf4Δ* is consistent with the results of the sensitivity test, as the growth of *snf4*Δ strains was completely abolished on medium supplemented with 0.8 μg/ml caspofungin, a β 1,3-glucan synthase inhibitor (Figure 3(a)). In addition, chromatography was performed to determine the sugar content of the fungal cell wall. The results showed that chitin, glucan and mannan in the cell wall exhibited significant reductions in the *C. tropicalis SNF4* deletion strains (Figure 3(d,e)).

### *SNF4* deletion promotes cell aggregation and pseudohypha formation in *Candida tropicalis*

**Figure 4.**
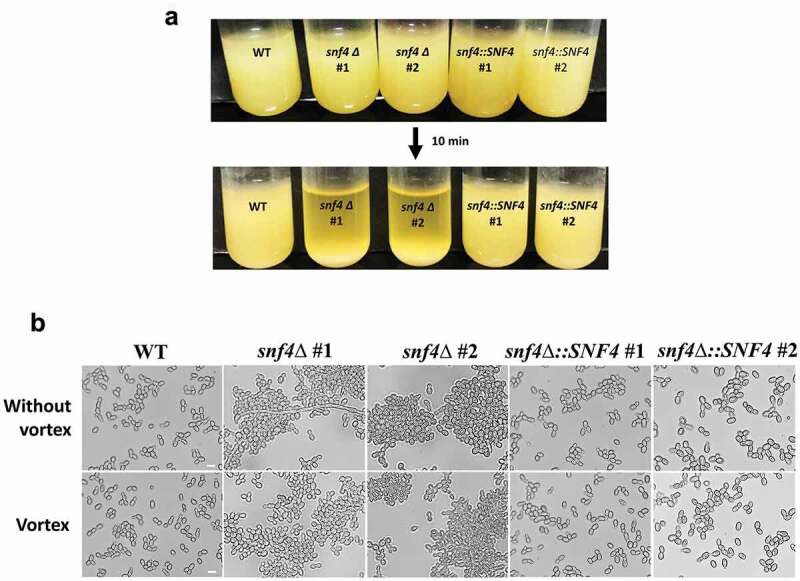
*C. tropicalis snf4δ* exhibited high cell sedimentation rates and cell aggregation. (a) Overnight YPD cultures showed that most *snf4δ* cells precipitated rapidly. (b) the representative images of precipitated samples showed severe aggregation of *snf4δ* cells. Scale bar: 5 µm.

We found that the *C. tropicalis snf4Δ* strains exhibited striking differences in sedimentation rates after measuring the ability of cells to fall through and accumulate together (Figure 4(a)). This phenomenon has not been reported or observed in *C. albicans snf4Δ* [22]. The data suggest a divergent role of Snf4 between the two *Candida* species and imply that *C. tropicalis* Snf4 might play an additional role in cell separation and cell‒cell interactions.

**Figure 5.**
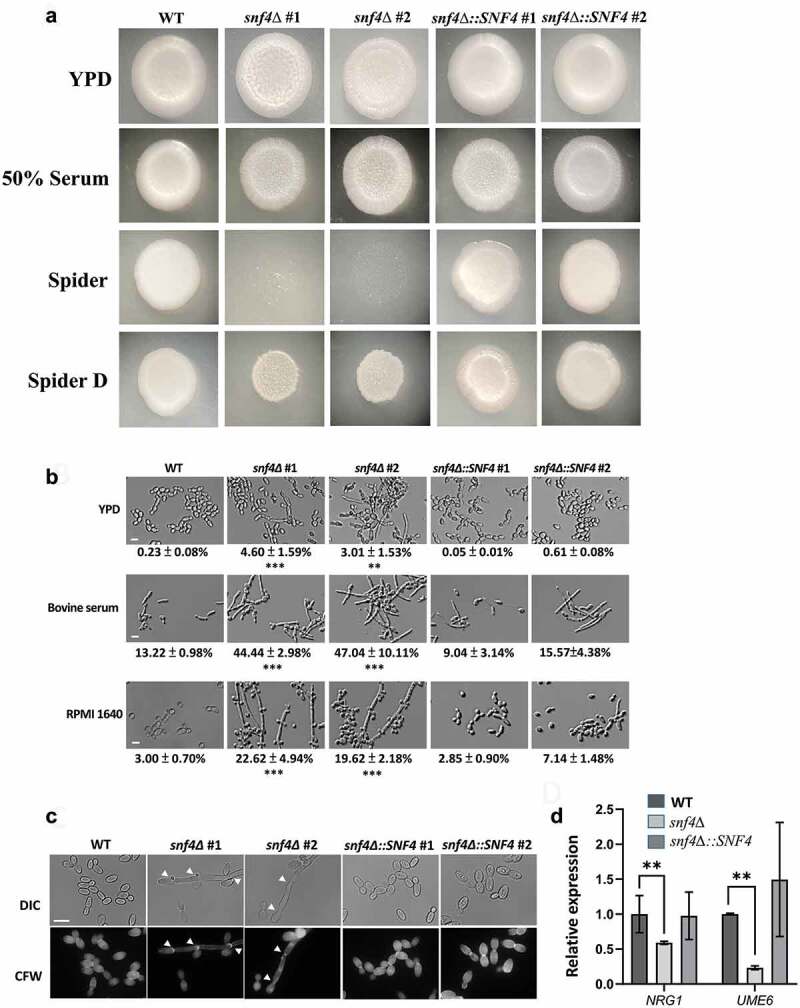
*SNF4* deletion strains promoted pseudohypha formation. (a) Colony morphologies of the wild-type, *snf4δ* and complemented strains. (b) Representative images showing that *snf4δ* in YPD, serum, and RPMI 1640 media exhibited significant increases in hypha formation. The ratios of hypha formation are displayed below each image. Scale bar: 5 µm. (c) Calcofluor white staining demonstrated that most filamentous cells in *C. tropicalis snf4δ* were pseudohyphal cells. White arrows indicate constriction sites. Scale bar: 5 µm. (d) *SNF4* deletion resulted in lower expression of *NRG1* and *UME6* in *C. tropicalis*. The values are the means ± SDs from three experimental replicates. **, *P*<0.01; ***, *P*<0.001.

Furthermore, compared to the wild-type and complemented strains, cells of two independent *snf4Δ* strains obtained from the aggregated cells formed few hyphae, as observed by microscopy (Figure 4(b)). The filamentous growth and hypha formation ability in different culture conditions was therefore tested on agar plates and quantified in liquid media. As shown in Figure 5(a), similar to *C. albicans* [22], the *snf4Δ* strains could not grow on Spider medium due to the presence of mannitol as the carbon source. Replacement with a low concentration of glucose (Spider D) partially restored its viability. Interestingly, two distinct phenotypes observed in *C. tropicalis snf4Δ* were different from those in *C. albicans* [22]. *C. tropicalis snf4Δ* still grew well in the YPD + serum medium and showed a wrinkling phenotype with some filamentous growth in YPD, YPD + serum and Spider D culture conditions (Figure 5(a)).

Additionally, *snf4Δ* strains exhibited significant increases in hypha formation in YPD, bovine serum and RPMI 1640 liquid medium (Figure 5(b)). The hyphae produced by the *snf4*∆ mutant strains were segmented and had chain-like cells (pseudohyphae), rather than elongated thread-like filament cells (true hyphae) (Figure 5(b)), and constriction sites could be easily distinguished after calcofluor white staining (Figure 5(c)). However, *C. albicans snf4Δ* showed no difference in hypha formation between the wild type and *snf4Δ* in YPD but exhibited decreases in filamentation in Spider medium and RPMI + serum liquid medium, although these data have not been quantified [22]. These data suggest that hyphal regulatory circuits and the characteristics of Snf4 differ between the two species. We further determined the expression of some hypha-associated genes. The hypha-repressed gene *NRG1* and the hypha-specific gene *UME6* were selected for testing. The major reason was that the expression level of *UME6* has been proposed to determine yeast, pseudohypha and true hyphal transitions [62]. Indeed, qPCR results showed a significant reduction in *NRG1* and *UME6* expression in the *snf4Δ* strain (Figure 5(d)).

### Deletion of *C.* *tropicalis SNF4* significantly reduced virulence and fungal burden in the greater wax moth and murine model

**Figure 6.**
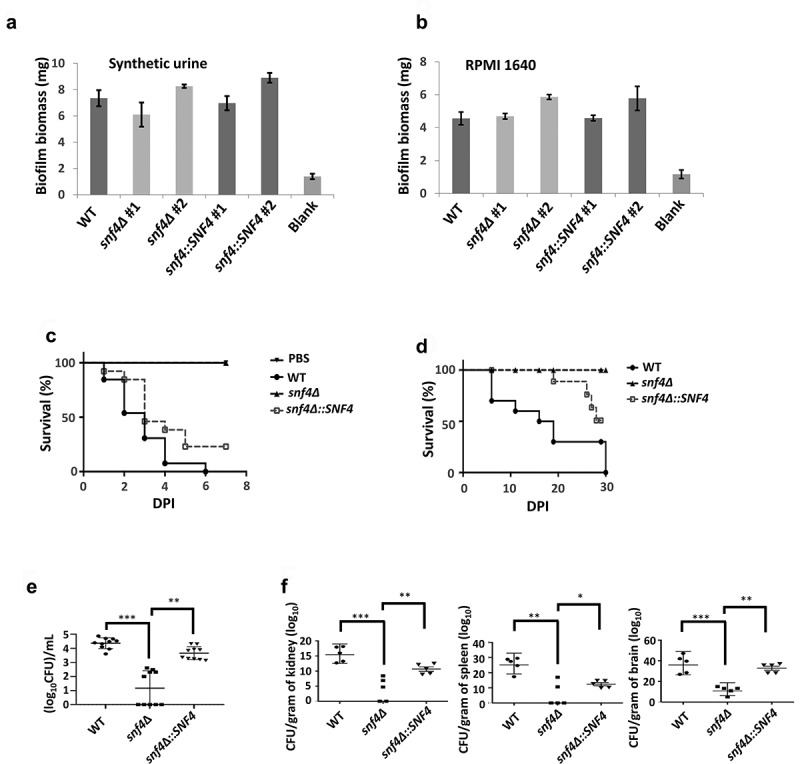
*SNF4* deletion strains did not affect biofilm formation but exhibited reduced virulence and resulted in lower fungal burdens. *SNF4* deletion strains exhibited a similar ability to form biofilms in (a) synthetic urine and (b) RPMI 1640 media compared to the wild-type and complemented strains. Survival curves of (c) wax moth (n = 10) after infection with 2.25 × 10^6^ *C. tropicalis* cells or (d) mice (n = 9) infected 5 × 10^6^ *C. tropicalis* cells showed that *snf4δ* is required for virulence. The fungal burden in (e) haemolymph of each wax moth (n = 10) and (f) each organ of *C. tropicalis*-infected mice (n = 5) was determined on the third day after infection. *, *P*<0.05; **, *P*<0.01.

Sugar sensing has profound effects on cellular physiology, including on cell growth, carbon metabolism, cell wall integrity, colonization and virulence, in *C. albicans* [20, 21–23]. Solid evidence regarding the abovementioned characteristics has been obtained for *C. albicans SAK1*, an Snf1-regulated gene [22]. However, the *C. tropicalis snf4Δ* strains exhibited increases in filamentation, completely opposite to the result observed for *C. albicans snf4Δ* [22]. In addition, *SNF4* deletion in *C. tropicalis* showed no biofilm defects in either synthetic urine or RPMI medium (Figure 6(a,b)). Although *snf4Δ* strains showed completely abolished biofilm formation in Spider medium, this was due to the inability to utilize mannitol as the sole carbon source for growth, leading indirectly to the inability to produce biofilms (data not shown). We therefore wondered about the impact of *SNF4* on virulence in *C. tropicalis*. As shown in Figure 6(c,d), compared to the wild-type strain and complemented strains, the virulence of the *snf4*∆ strain was significantly reduced in both the greater wax moth and the murine models. Additionally, the *snf4*∆-infected *G. mellonella* and mice exhibited a drastic drop in fungal burden in the *G. mellonella* haemolymph, and spleens, kidneys and brains, respectively (Figure 6(e,f)).

## Discussion

Carbon sensing and metabolic flexibility are crucial for *C. albicans* to maintain cellular physiology and assist in host colonization and infection [20, 21–23]. Central to understanding the dynamic sensing of carbon sources by *C. albicans* is AMPK, via which cells can detect changes in environmental glucose levels and adapt to alterations [12]. Specifically, under conditions with sufficient glucose, yeasts preferentially metabolize glucose while inhibiting the metabolism of nonglucose carbon sources at the same time. This phenomenon is called glucose repression [63]. However, low glucose levels could activate the AMPK pathway, thereby reactivating the metabolism of the nonglucose carbon sources (glucose derepression), allowing *C. albicans* to survive under insufficient glucose [20, 21, 22, 45]. Nevertheless, the role of the *SNF4* gene in *C. tropicalis* remains unclear and was investigated here. Our study shows convergent and divergent roles of *SNF4* between *C. albicans* and *C. tropicalis*. In particular, in the two species, *SNF4* is required for activation of the Snf1/AMPK pathway and is involved in glucose sensing and metabolism of nonglucose carbon sources, cell wall integrity and virulence potential, although the pathogenesis of *C. albicans SNF4* has not been investigated. However, *snf4*∆ exhibited striking differences in cell aggregation and hypha formation between the two *Candida* species, wherein *C. tropicalis snf4*∆ showed marked defects in cell separation and filamentous behaviours, different from those of *C. albicans* [22].

Mechanisms underlying hyphal growth in *C. albicans* are controlled by several MAPK and cAMP pathways with many hypha-specific genes [64–69]. Hypha formation can be induced by high temperature, CO_2_, serum and GlcNAc [25, 70–73]. However, studies regarding hypha formation mechanisms are very limited in *C. tropicalis*, although this morphological transition has been observed during incubation with Lee’s medium and serum [27, 74]. Interestingly, completely different from the result for *C. albicans*, supplementation with GlcNAc inhibits hyphal growth in *C. tropicalis* [72, 75], indicating that the intrinsic features of hyphal growth between the two *Candida* species are different and depend on the types of nutrition. Several studies have further demonstrated that the regulatory networks involved in hypha formation of the two pathogens could be divergent [27–31]. Similarly, we found that *SNF4* acts as a repressive regulator in hypha formation in *C. tropicalis* but acts as a positive regulator in *C. albicans* [22]. Furthermore, *snf1*∆ and *mig1*∆/*mig2*∆ double mutants in budding yeast resulted in impaired pseudohyphal filamentous growth [76]. The reasons why taxonomically related *Candida* species with conserved AMPKs exhibit markedly divergent roles in controlling hypha formation remain unclear.

Nrg1, a hyphal repressor, and Ume6, a hyphal enhancer, function together to regulate the yeast-hyphal transition [77–82]. Interestingly, the expression levels of *NRG1* and *UME6* were significantly reduced in the *C. tropicalis snf4*∆ strains. Previous studies have shown that *UME6* deletion strains were unable to form true hyphae but retained the ability to form pseudohyphae in *C. albicans* [62, 77, 82, 83]. Hence, we assume that low expression of *NRG1* and *UME6* might promote pseudohypha formation in *C. tropicalis*.

The cell wall acts as the outermost protective layer of the fungal cell, in addition to maintaining the shape of the cell and protecting the cell from external stress such as that imposed by drugs or the host immune system [53, 55, 84–86]. Meanwhile, the fungal cell wall is also regarded as an important target for the development of antifungal drugs [87–89]. Similar to budding yeast [90, 91], we observed that the *C. tropicalis snf4Δ* strains were highly sensitive to caspofungin and calcofluor white, indicating that AMPK is required for cell wall strength. Indeed, the decline in the expression level of the *FKS1* and *FKS2* genes (encoding β-1,3-glucan synthase) and the significant reduction in the glucan content of the cell wall in the *C. tropicalis snf4*∆ strains can explain why the mutant strains are highly susceptible to caspofungin. Furthermore, the lower the chitin content is, the higher the sensitivity to calcofluor white [51], which is consistent with results regarding the carbohydrate content in the cell wall; however, after normalization with *ACT1* or *RDN18*, different outcomes were observed for the quantitative expression level of *CHS1*. This could be explained by the lower stability of *ACT1* expression, as several studies have recorded the fold-change in *ACT1* expression during treatments [77, 92–94]. The inconsistent results between chitin gene expressions and chitin content suggest that the posttranscriptional mechanisms might occur in this scenario. Additionally, five mannan genes were significantly reduced in the *snf4Δ* strain and might directly impact mannosylation in *C. tropicalis*. Finally, the significant reduction in glucan and chitin levels might indirectly decrease the content of mannan in *C. tropicalis snf4*∆ cells since the inner layer of the cell wall (glucan and chitin) is the supporting scaffold for the outer layer of the cell wall (mannan) [53,54]. Nevertheless, the reduction in chitin and mannan levels could also be due to the inhibitory effect of other metabolic genes involved in chitin and mannan synthetic pathways.

*C. tropicalis snf4Δ* exhibited high sensitivity to SDS and fluconazole. We assume that loss of *snf4Δ* resulting in an insufficient protective layer of the cell wall could cause easier cell membrane exposure, leading to increased sensitivity to cell membrane-perturbing agents. It is also possible that changes in cell wall integrity alter cell membrane structure, composition and properties. Nevertheless, the exact roles of *SNF4* in mediating the function of the *C. tropicalis* cell membrane remain unknown.

The cell wall is also an important structure for environmental surface and cell‒cell interactions [54]. The striking defects in the cell wall composition of *C. tropicalis snf4∆* strains resulted in severe cell aggregation. Interestingly, the phenomena found in *C. tropicalis snf4∆* were not observed and reported for *C. albicans snf4∆* [22]. Whether the marked alterations in the cell wall composition of *C. tropicalis snf4∆* cause cell aggregation requires further investigation.

Several reports have demonstrated that AMPK plays a crucial role in delaying ageing in eukaryotic species [36, 46–48]. In particular, caloric restriction (CR) can extend longevity in budding yeast through the AMPK pathway [36]. Similarly, the CR longevity response is mediated by *C. tropicalis* AMPK, as *SNF4* deletion severely affected CLS extension. In budding yeast, CR-induced CLS extension is mediated by Cat8 (CATabolite repression), a zinc cluster transcription factor required for the activation of many carbon source-responsive elements (CSREs) [95]. Two potential orthologs of *CAT8* genes were found in *C. tropicalis*, *CTRG1_03210* and *CTRG1_02593*, which are orthologs of *CAT8* and *ZCF16* (former name: CAT8) of *C. albicans* [33], as well as orthologs of *CAT8* and *SIP4* (C6 zinc factor required for CSRE activation) in budding yeast [95, 96]. It would be interesting to further explore the CLS mechanisms of the AMPK pathway in *Candida* species.

We showed that *snf4*∆ strains had significantly increased pseudohyphal filamentous growth. Furthermore, biofilms of the *snf4*∆ strains were similar to those of the wild-type strain, and hyphae, pseudohyphae and biofilms are important for virulence [62, 68, 83, 97–99]. However, two different infection models showed that *C. tropicalis SNF4* is crucial for pathogenesis. It is possible that infected hosts have complex environments that may lead to different outcomes in hyphal development. Furthermore, the changes in the cell wall composition of *C. tropicalis snf4*∆ might directly affect cell survival, and alterations in cell wall polysaccharides could have a considerable impact on innate immune recognition [53,100–102], with direct consequences for pathogenesis. Thus, we assumed that the *C. tropicalis snf4*∆ cell itself tends to activate the pseudohyphal regulatory network to scavenge as many nonglucose carbon nutrients as possible for survival [103], rather than for invasion.

In summary, similar to that in *C. albicans*, AMPK in *C. tropicalis* is responsible for the metabolism of nonglucose carbon sources and is involved in cell wall integrity and virulence. Interestingly, some phenotypes, such as cell aggregation and pseudohypha formation in *C. tropicalis snf4*∆, have not been reported or observed in *C. albicans ssk1*∆ and *snf4*∆ [22]. These data indicate the convergent and divergent roles of the Snf1-Snf4 AMPK in *C. tropicalis*.

## Data Availability

The authors confirm that the data supporting the findings of this study are available within the article.
